# Influence of pharmacological preconditioning on the results of lifting operations efficiency

**DOI:** 10.1016/j.heliyon.2018.e00758

**Published:** 2018-08-30

**Authors:** N.E. Manturova, V.A. Stupin, G.O. Smirnova, E.V. Silina

**Affiliations:** a Pirogov Russian National Research Medical University (RNRMU), Ostrovityanova St. 1, Moscow, 117997, Russia; b Institute of Plastic Surgery and Cosmetology, Olkhovskaya St., 27, Moscow, 105066, Russia; c I.M. Sechenov First Moscow State Medical University (Sechenov University), Trubetskaya St., 8, Moscow, 119991, Russia; d Kaluga Regional Clinical Hospital, Vishnevskaya St., 1, Kaluga, 248007, Russia

**Keywords:** Medicine, Surgery

## Abstract

The main *aim* of the study is to determine the effectiveness and safety of lifting operations in women with varying degrees of involuntary changes of facial skin, in particular when applying pharmacological conditioning, with the objectification of the role of the latter.

**Materials and methods:**

A research and surgical treatment were conducted to eliminate involutional changes of various degrees in the facial skin of 461 women aged 35–75 years. Surface lifting was performed in 20.2% of patients, SMAS-lifting – 49.0%, SMAS-lifting with a three-level endoscopic assist lift of the lower face zone was performed in 30.8% of women. Before the surgery in 13.2% of cases, I degree of involutional changes in facial skin was registered, 47.9% – grade II, 38.9% – grade III. Patients were divided into two comparable groups. With the standard preparation without additional drug correction, 299 women (64.9%) were operated on in the preoperative period, they made up a comparison group. The main group included 162 (35.1%) women who underwent therapeutic conditioning before the lifting operation (Cytoflavin, n = 86; Actovegin n = 23; Ethylmethylhydroxypyridine succinate, n = 32; Meldonium, n = 21; Pentoxifylline, n = 31; Vinpocetine n = 27). Instrumental evaluation of the skin dermal microcirculation was performed using laser Doppler flowmetry and estimation of transcutaneous oxygen tension. In the blood plasma, the parameters of free radical processes (FRP) were studied. FRP were studied in terms of generation of active oxygen forms by leukocytes – intensity of chemiluminescence basal and intensity of chemiluminescence stimulated, as well as antiperoxide plasma activity and malondialdehyde. Early postoperative complications were analyzed, the number of repeated lifting surgical corrections on the face was studied for 5 years.

**Results:**

The role of FRP in the pathogenesis of involuntary changes in the facial skin has been established. The imbalance of FRP was expressed in the intensification of the reactive oxygen species generation and products of lipid peroxidation. This correlated with disorders of cutaneous microcirculation and a decrease in the saturation of the facial tissues with oxygen, manifested by an increasing energy deficit and the severity of involutional skin changes. The obtained data justify the expediency of using pharmacological conditioning with energy correcting antioxidant medicine. Preoperative conditioning allowed to reduce the number of early postlifting complications associated with tissue trophism in a quarter, especially during surface lifting. In addition, in the preconditioning group, the scar was more cosmetic already at the seventh day after the operation. Based on the study of postoperative catamnesis, self-assessment data and laboratory-instrumental methods of skin system examination in people of different ages, it was revealed that while using SMAS-lifting with a three-level endoscopic-assisted lifting of the lower part of the face, the lowest frequency of complications and the best 5-year effectiveness were established.

## 1 Introduction

The significant aging in the population of economically developed countries with the increase in the share of older people in its structure causes a legitimate interest in studying the mechanisms of aging [1, 2, 3, 4], including involutional changes in the skin system [5, 6, 7, 8]. Improving the living standards of mature and advanced age people, including the preservation and restoration of aesthetic human health, is considered today as one of the urgent tasks of medicine, since the appearance of an older person is an equally necessary component of the communicative social function performance. Appearance is possibly even has more influence on social well-being, social adaptation and quality of life of a person than the state of other bodies.

As the population ages, the number of procedures performed by cosmetologists and plastic surgeons is increasing exponentially in a geometric progression [9, 10, 11]. On the one hand, the advantages of surgical treatment is a radical approach to correct the external manifestations of age-related changes. But on the other hand, the greatly increased interest in surgical methods for correcting involutional changes in the facial skin is far from always justified by real clinical successes [12, 13]. Moreover, many involuntary changes can be slowed down with not so aggressive treatment methods, sanogenesis of which is not fully used in modern clinical practice of aesthetic medicine.

Fundamental science has revealed the main pathogenetic mechanisms of skin aging, which include involuntary changes in the system of its blood supply. In addition, the role of free radical processes (FRP), which lead to damage to all of its cellular elements during aging, is repeatedly noted [14, 15, 16, 17]. This allows to formulate new therapies based on the correction of these pathophysiological components of aging processes, which includes an improvement of the plastic surgery operations efficiency.

In this connection, a study was carried out to determine the efficacy and safety of lifting operations in women with varying degrees of involuntary changes in the facial skin, including the use of pharmacological conditioning, with the objectification of the role of the latter.

## 2 Materials and methods

A survey and surgical treatment to eliminate involutional changes in the facial skin of various degrees in 461 women aged 35–75 years were conducted. 41 (8.9%) patients at the time of surgery was younger than 41 years, 226 (49.0%) – aged 41–55 years and 194 (42.1%) – over 55 years. The severity of the involutional changes in the facial skin varied from I to III degree (I degree – visualized superficial wrinkles within the epidermis, mimic wrinkles partially disappear in the absence of facial expressions, a slight change in the face oval, sagging of the soft tissues below the edge of the lower jaw to 0.5 cm. II degree – single or multiple group wrinkles with skin folds, covering the epidermis and dermis, mimic wrinkles are not smoothed with a calm expression, the presence of vertical skin folds in the parotid region, sagging of upper and lower eyelid skin, moderate deformation of facial oval, sagging of soft tissues below the edge of lower jaw by 0.5–1.5 cm. III degree – deep single or multiple wrinkles extending to entire skin in the form of chaotically located deep furrows, constantly, the presence of vertical folds in the chin region, neck, doubling skin folds of the lower eyelids, severe deformation of the face oval, sagging of the soft tissues below the edge of the lower jaw more than 1.5 cm). In 13.2% of cases, I degree of involutional changes in facial skin was registered, 47.9% – grade II and 38.9% – grade III.

Instrumental assessment of the skin condition was carried out using laser Doppler flowmetry (LDF) (BLF-21 single-channel instrument manufactured by Transonic System Inc., USA), and with the use of transcutaneous oxygen tension (TCM 400 device from RADIOMETER, Denmark). To assess the state of free radical processes (FRP) in the blood plasma of 84 operated women before the operation, the following laboratory parameters were investigated. Generation of reactive oxygen species (ROC) was determined using a chemilumenometer according to the indices – intensity of chemiluminescence basal (ICb) and intensity of chemiluminescence stimulated by zymosan (ICs), expressing the final result in mV/sec × 10^6^ white blood cells. The marker of lipid peroxidation – malondialdehyde (MDA), as well as the antiperoxide activity of plasma (APA) – the ratio of spontaneous chemioluminescence of secondary plasma to hydrogen peroxide induced chemioluminescence of secondary plasma was studied. FRP was also studied in 21 women aged 20–30 years inclusive, who did not have any involuntary changes in the skin of the face and neck (control group).

To correct the involutional changes in the facial skin, the patients after the complex clinical, instrumental and laboratory examination performed the following operations of facial and neck skin to correct involutional changes: surface lifting, SMAS-lifting and SMAS-lifting with a three-level endoscopic assist lift of the lower face zone. The standard movement of the facial skin involves the allocation of skin-fat flap within three large zones (temporal, buccal and postaural) with excision of excess skin. In this case, the tightening of the skin is performed through a wide operational access. SMAS-lifting pushes a frame of the classic facelift correction by raising subcutaneous fat and the hypodermic neck muscle with the neck skin as a complex wide musculocutaneous flap. SMAS-lifting with a three-level endoscopic assist lift of the lower face zone is a layer-by-layer tightening of all layers of the skin and muscles through punctures and performing part of the operation by the technique of video endoscopy through small cutaneous accesses. Superficial lifting was performed in 93 (20.2%) cases, including 19 (20.4%) women under 41 years of age, 57 (61.3%) – aged 41–55 years and 17 (18.3%) women over 55 years of age. SMAS-lifting was performed in 226 (49.0%) women, 112 (49.6%) of whom were older than 55 years, 92 (40.7%) – 41–55 years, 22 (9.7%) women on the time of the operation was less than 41 years. Implemented in recent years to the practice of the aesthetic surgery SMAS-lifting with a three-level endoscopic assist lift of the lower zone of the face, was performed in 142 (30.8%) women, including 77 (54.2%) women aged 41–55 years and 65 (45.8%) are over 55 years old.

Enrolled in the study, the patients were divided into two comparable groups. With the standard preparation without additional drug correction, 299 women (64.9%) were operated in the preoperative period, they made up a comparison group (group I). The main group (II) consisted of 162 (35.1%) patients who for an average of 10 days (from 7 to 14 days) before the surgery was prescribed therapeutic pre-operative conditioning, i.e. pharmacological correction of pathophysiological components of involutional skin changes, which included the effect on tissue metabolism disorders by stimulants of aerobic and anaerobic glycolysis and tissue perfusion. In some patients, this therapy was carried out not only in the pre- and early postoperative period, but in a number of cases, based on the clinical and laboratory examination, was extended to 6 months after discharge. An antioxidant and energy corrector Cytoflavin (succinic acid + inosine (riboxin) + nicotinamide + riboflavin sodium phosphate (riboflavin)) was administered to 86 women (54.9%) in the main group II at a dose of 1–2 tablets (425 mg) × 2 times a day. In addition, antioxidants Actovegin (n = 23, 14.2%) at a dose of 200 mg × 3 times a day, Ethylmethylhydroxypyridine succinate (n = 32, 19.8%) in a dose of 125 mg 3 times a day, Meldonium (n = 21; 13.0%) in a dose of 300 mg × 2 times a day. In addition, Pentoxifylline was administered to 31 patients (19.1%) at a dose of 100 mg × 3 times a day, Vinpocetine (n = 27, 16.7%) at a dose of 10 mg × 3 times a day as correctors for microcirculatory blood flow. 58 (35.8%) patients received two of the above mentioned medicines, more often sequentially. The most frequently were used combinations with Cytoflavin (n = 27), among which Cytoflavin and Pentoxifylline were used more often (n = 9). Groups I and II were comparable in age and surgical correction methods (Table 1).

**Table 1:** Characteristics of groups of patients by age composition and operations.

	The comparison group (I)	The main group (II)	p	In all
N	299	162		461
Age				
35–40 years	30 (10.0%)	11 (6.8%)	0.056	41 (8.9%)
41–55 years	155 (51.8%)	71 (43.8%)		226 (49.0%)
Over 55 years	114 (38.2%)	80 (49.4%)		194 (42.1%)
Operations				
Superficial lifting	55 (18.4%)	38 (23.5%)	0.223	93 (20.2%)
SMAS-lifting	155 (51.8%)	71 (43.8%)		226 (49.0%)
SMAS-lifting with a three-level endoscopic assist lift of the lower zone of the face	89 (29.8%)	53 (32.7%)		142 (30.8%)

To evaluate the efficacy and safety of operations, early postoperative complications recorded within 7 days after surgery were analyzed, and long-term efficacy results were studied in 5 years after the lifting operation, mainly, the number of repeated lifting surgical corrections on the face was studied for 5 years. Long-term results could be traced in 245 (53.2%) – (in group I, n = 148 (49.5%), in group II n = 97 (59.9%)).

**Statistical processing** of data was carried out using the software SPSS 17.0 with the use of parametric and nonparametric criteria for assessing statistical significance. Differences were considered significant at p < 0.05. Descriptive statistics of qualitative parameters are presented in the form of frequencies (abs, %), quantitative – median (Me), lower (25%) and upper (75%) quartile in cases where the parameter had a distribution function far from normal. To compare the two independent nonparametric samples, the Mann-Whitney test was used, for a multiple comparison, the Kruskal-Wallis test. Qualitative variables were compared using the χ^2^ test (Pearson XY-square, conjugacy table analysis). The correlation analysis was performed by the Pearson method.

## 3 Results

The results of the FRP study objectified their role in the development of involuntary age-related changes in the facial skin. At the first stage of the study, a comparative analysis of various parameters of FRP was performed in operated patients with the same parameters of women in the control group who did not have any involuntary changes in the skin of the face and neck. Significant differences in the parameters of ICb and ICs were established. Thus, the ICb indicator in the control group was, on average, 1.96 times higher than before the operation in patients with involuntary skin changes of varying degrees (p < 0.05). The ICs, on the contrary, in the operated patients was 1.95 times higher than in the control group (p < 0.05). Despite the fact that the level of APA and MDA were characterized by a tendency to increase in comparison with control, these indicators were statistically comparable in both groups (Table 2).

**Table 2:** Parameters of free-radical processes in operated patients in comparison with the control group.

	ICb (mV/sec X 10^6^ leucocytes)	ICs (mV/sec X 10^6^ leucocytes)	APA	MDA (μmol/l)
The control group (n = 21)	74.4 [52.4; 87.8]	399.7 [272.6; 580.1]	2.19 [1.7; 3.1]	2.56 [2.5; 2.8]
Operated patients (n = 84)	38.0 [19.2; 72.2]	777.8 [469.9; 1258.0]	2.46 [1.9; 3.1]	2.83 [2.1; 3.2]
р (Mann-Whitney test)	0.003	<0.0001	0.473	0.162

*Note*: statistical results are presented in the form of the following data: the first line is the median (Me), the second line is the lower and upper quartiles [Quartile 25%; Quartile 75%].

The next stage of the study was a comparative analysis of the FRP indices in women at various degrees of involuntary changes in the facial skin system (Table 3). A statistically significant regression of ICb has been established as the degree of involuntary changes in facial skin progresses. Thus, in patients with the first degree of involuntary changes in the facial skin, the ICb was 1.35 times greater than at grade II (p < 0.05), and 1.87 times higher than in patients with grade III (p < 0.05), and at II degree ICb was 1.38 times higher than at grade III (p < 0.05). The ICs, on the contrary, increased in proportion to the degree of involuntary changes. In patients with grade III, ICs was 2.01 times greater than at grade I (p < 0.05) and 1.53 times higher than in patients with grade II involuntary changes in facial skin (p < 0.05), and at grade II, the ICs was 1.32 times higher than at grade I (p < 0.05).

**Table 3:** Comparative analysis of FRP indicators in women at different degrees of involuntary changes in the facial skin system.

Factor	I grade (n = 20)	II grade (n = 29)	III grade (n = 35)	р (Kruskal-Wallis)
ICb (mV/sec × 10^6^ leucocytes)	59.3 [35.2; 80.4]^∗^^,^^1/2, 1/3^	43.8 [19.3; 76.4]^∗^^,^^1/2, 2/3^	31.7 [12.9; 67.2]^∗^^,^^1/3, 2/3^	0.017*
ICs (mV/sec × 10^6^ leucocytes)	471.7 [295; 982]^∗^^,^^1/2, 1/3^	620.7 [414; 1227]^∗^^,^^1/2, 2/3^	948.3 [677; 2968]^∗^^,^^1/3, 2/3^	0.009*
APA	2.24 [1.83; 2.53]	2.41 [1.90; 3.19]	2.60 [1.67; 3.44]	0.189
MDA (μmol/l)	2.67 [1.9; 3.0]^∗^^,^^1/3^	2.81 [2.1; 3.4]	3.24 [2.0; 3.6]^∗^^,^^1/3^	0.043*

*Note*: statistical results are presented in the form of the following data: the first line is the median (Me), the second line is the lower and upper quartiles [Quartile 25%; Quartile 75%].

*p < 0.05 – significant intergroup difference. Comparison of 3 independent groups according to the Kruskal-Wallis test; comparison of 2 groups – according to the Mann-Whitney test (^1/2^ I and II degrees, ^1/3^ I and III degrees; ^2/3^ II and III degrees).

Changes in the oxygen phase of the FRP in the form of a decrease in ICb and the growth of ICs caused changes in lipid markers in the blood of women with severe involuntary skin changes. The APA index was characterized by a tendency to increase as the involuntary changes in the skin system progressed. The difference in the APA index between groups with I and III degrees was 1.15 times (p > 0.05). The MDA in women increased in proportion to the degree of involuntary processes and averaged 2.67; 2.81; 3.24 μmol/l for I, II, III degrees of involuntary skin changes, respectively. And in patients with grade III MDA was significantly 1.21 times higher than at grade I (p < 0.05). Significant differences in MDA in patients with I and II degrees, as well as II and III degrees of involuntary changes in the skin were not established.

According to laser Doppler flowmetry (LDF) it was established that the microcirculation of the facial skin decreased significantly as the degree of involutive skin processes increased. The mean total LDF values (including the total quantitative representation of blood flow in all studied points) in women with the first degree of involuntary changes in facial skin were 1.20 times higher than in patients with grade III (p < 0.05). The indicators of transcutaneous oximetry also decreased as the degree of involuntary changes in the facial skin increased. TcpO_2_ in women with I degree were on average 1.38 times higher than in patients with grade III (p < 0.05) (Table 4). It is important to note that in the control group, LDF and TcpO_2_ were significantly higher than those in the patients undergoing surgery. LDF in adolescent, not having any manifestations of involutive changes, averaged 8.0 ml/min (interquartile interval [6.4, 9.3] ml/min), TcpO_2_ – 54 mm Hg (interquartile interval [47; 65] mm Hg).

**Table 4:** Indices of tissue oximetry and microcirculation of the facial skin in patients with varying degrees of involutional changes in the facial skin system.

Factor	I grade (n = 20)	II grade (n = 29)	III grade (n = 35)	Р (Kruskal-Wallis)
LDF, ml/min	7.7 [5.4; 9.0]^∗^^,^^1/3^	7.1 [5.2; 8.1]	6.4 [5.0; 7.8]^∗^^,^^1/3^	0.019*
TcpO_2_, mm Hg	47 [41; 57]^∗^^,^^1/3^	40 [34; 49]	34 [30; 41]^∗^^,^^1/3^	0.010*

*Note*: statistical results are presented in the form of the following data: the first line is the median (Me), the second line is the lower and upper quartiles [Quartile 25%; Quartile 75%].

*p < 0.05 – significant intergroup difference. Comparison of 3 independent groups according to the Kruskal-Wallis test; comparison of 2 groups – according to the Mann-Whitney test (^1/2^ I and II degrees, ^1/3^ I and III degrees; ^2/3^ II and III degrees).

A direct correlation relationship between LDF and ICb (r = 0.315, p < 0.05) was established, and LDF-inverse correlation with the ICs (r = −0.392, p < 0.05) was found.

The obtained data made it possible to clarify the role of FRP imbalance in pathophysiological mechanisms of aging of the skin system. Imbalance FRP, coupled with tissue hypoxia and a decrease in tissue blood flow, explains the advisability of early energy-based antioxidant pharmacological preconditioning. Therefore, the next stage of the study is devoted to assessing the safety and effectiveness of ongoing operations to eliminate involuntary skin changes, as well as assessing the impact of energy-saving therapy.

The total number of early postoperative complications was 37 (8.0%). After the superficial lifting of the cases of early postoperative complications, there were 10 (10.8%), after SMAS-lifting – 25 (11.1%), after SMAS-lifting with three-level endoscopic lifting of the lower zone of the face – 3 cases (2.1%). This speaks in favor of performing SMAS-lifting with a three-level endoscopic lifting of the lower zone to correct involute changes in the facial skin.

In the main group of patients receiving energy-corrective therapy, the total number of complications was 11 (6.8%), in the comparison group – 26 (8.7%), which is 1.28 times bigger (p < 0.05). The obtained data indicate that preoperative conditioning allows to reduce the number of early postlifting complications in a quarter. This was best manifested in the course of surface lifting (complications in the main group were only 2.6%, and in the comparison group – 16.4%, 6.3 times more). The least early postoperative complications were after SMAS-lifting with a three-level endoscopic of the lower zone of the face (3.4% in the comparison group and 0% in the main group). Analysis of early postoperative complications in patients of both groups is presented in Table 5.

**Table 5:** Complications in the early postoperative period in patients with and without preoperative conditioning.

Complications	Operations	Superficial lifting (n = 93)		SMAS-lifting (n = 226)		SMAS-lifting with a three-level endoscopic assist lift of the lower face zone (n = 142)		Patients in all (n = 461)	
Groups		I	II	I	II	I	II	I	II
Hematomas		4 7.3%	0	8 5.2%	9 12.7%	0	0	12 4.0%	9 5.6%
Hair thinning/hair loss		2 3.6%	0	3 1.9%	0	2 2.2%	0	7 2.3%	0
Marginal necrosis		2 3.6%	0	2 1.3%	0	1 1.1%	0	5 1.7%	0
Paresis of mimic muscles		1 1.8%	1 2.6%	1 0.7%	1 1.4%	0	0	2 0.7%	2 1.2%
In all		9 16.4%	1 2.6%	15 9.7%	10 14.1%	3 3.4%	0	26 8.7%	11 6.8%

Groups: I – without preoperative conditioning (comparison group), II-with preoperative conditioning (main group).

The most common complication after the lifting operation was a subcutaneous hematoma, diagnosed within the first 24 hours after surgery. An unreliable increase in the number of hematomas with open SMAS-lifting in the group of preoperative conditioning is explained by an active pharmacorrection that stimulated tissue blood flow (p > 0.05). The use of an auxiliary endoscopic technique allowed to completely avoid this type of complications. In none of the cases hematoma increased and did not require re-examination of the postoperative wound with the aim of definitively stopping the bleeding. With the development of hematomas, it was emptied through the hole between the stitches of the cutaneous suture, prescribed hemostatic medication and physiotherapeutic procedures were prescribed from 3 days postoperatively.

The frequency of paresis of the mimic muscles, resulting from the direct intersection of branches of the facial nerve or compression by retention suture and with deep anesthesia, was insignificant (0.9%) and did not differ statistically significantly in the groups analyzed. It should be noted that 2 patients out of 4 who developed this complication already had a history of surgery to eliminate involutional changes in the facial skin. Nerve conduction disorders were restored in the period of about 2 weeks, patients were prescribed physiotherapeutic procedures (electrophoresis with lidase, potassium iodide, magnetotherapy, laser therapy).

Marginal cutaneous necrosis along the suture line was observed in all operations with standard preoperative preparation and was completely absent in the preoperative conditioning group. Their frequency was 1.7%, with the majority of complications of this type (3.6%) detected during surface lifting, and with SMAS-lifting, the frequency of such complications was 1.2%. These complications were associated with excessive tension of the skin flap in conditions of impaired tissue trophism. In the SMAS-lifting group with pharmacotherapy this complication was not revealed due to controlled skin tension and better blood supply to the SMAS flap. With the development of this complication, energy-correcting pharmacotherapy and physiotherapy were applied from 1 day postoperative period to soften the necrosis zone and more quickly wound healing.

Hair thinning and partial hair loss was observed in 7 cases, all of which occurred in patients without preoperative conditioning. This complication is a consequence of the disturbance of trophic hair follicles in the frontal-parietal and occipital areas, so stimulation of tissue blood flow and metabolic processes allowed them to be prevented.

Thus, a comparative assessment of early postoperative complications frequency has convincingly shown that preoperative conditioning reduces the number of complications associated with trophic tissue disorders.

Assessment of the scar status in 7 days after the lifting operation established that the postoperative scar was more cosmetic in the pharmacological preconditioning group (10–14% less density, size and infiltration of the postoperative scar).

The positive results obtained during preoperative conditioning convinced us and our patients in the advisability of continuing this approach to the correction of tissue disorders that promote the inhibition of the progression of involutional skin changes, including in the postoperative period. The next stage of our work was a long-term evaluation (after 5 years) of various surgical interventions results.

Evaluation of repeated operations to eliminate involuntary changes in skin of the face in 5 years after discharge showed that during the indicated period, there were 8.2% of cases (20 of 245 women who were observed for 5 years). 5 years ago, these patients underwent various types of surgical anti-aging interventions. 16 of them were after surface lifting, 3 – after SMAS-lifting, 1 – after SMAS-lifting with a three-level endoscopic assist lift of the lower part of the face. In the standard treatment group, there were 15 (10.1% of 148 women), in the conditioning group – 5 (5.2% of 97). All these patients were over 50 years old.

## 4 Discussion

The results of the study demonstrated the role of FRP imbalance in the pathophysiology of the skin system aging. Thus, the indicator of ICb, characterizing the level of intracellular FRP with which the leukocyte reacts to even the minimal oxygen deficiency that develops in the tissues, which provokes a decrease in the tissue renovation processes, normally is high and, with a favorable course of local metabolic reactions, remains so. If this increase is sufficient to solve the anti-inflammatory and antigenerative problems of aging skin, then the body cope with involuntary changes on its own. If there is not enough energy for the cellular elements, then the decrease in ICb is a marker of disorganization and development of an interstitial metabolic processes imbalance. This provokes a slow increase in dismetabolic processes, which are realized by involuntary skin changes especially in patients older than 50 years. The indicator of ICs reflecting the potential ability of the body to generate cytokines in response to the pathological process associated with tissue hypoxia, inflammation and ischemia, on the contrary, increases in proportion to the degree and severity of the involution processes of the facial skin system. As a result of these changes, the APA index, reflecting the level of response of the body's antioxidant systems, prevents the build-up of the damaging effect of oxidative stress, and the MDA index, a secondary product of free radical lipid peroxidation, reflecting the activity of the lipid cascade of oxidative stress in aging women.

The resulting changes in the unbalance of all stages of the FRP with the hyperactive production of reactive oxygen species and the growth of lipid peroxidation products in proportion to the degree of involutionally altered tissues are explained by the presence in women of older age who have a third degree of involuntary changes in the facial skin system, tissue hypoxia, and a decrease in tissue blood flow. These effects were indicated by instrumental methods. In the course of the study based on laser dopplerography and transcutaneous oxygen tension in women, a cellular energy deficit was established due to a decrease in blood flow and transcutaneous oxygen level. The TcpO_2_ and LDF values correlating with the FRP decrease as the degree of the involutive processes of the skin system increases.

Thus, imbalance of FRP, coupled with tissue hypoxia and lowering of tissue blood flow, explains the expediency of early energy-correlating antioxidant preconditioning.

Pharmacological preconditioning positively affected the results of lifting operations effectiveness. Thanks to pharmacological preoperative conditioning, it was possible to reduce the share of early postoperative complications from 8.7% to 6.8%, and the state of postoperative scars was improved. In addition, the group of energy correction noted a tendency to reduce the number of repeated operations to eliminate involuntary changes in the facial skin for 5 years, which is another important argument in favor of the pharmacological conditioning of various lifting operations.

Regarding the type of lifting operations, the advantage of SMAS-lifting with a three-level endoscopic-assisted lifting of the lower part of the face was demonstrated over the surface lifting and standard SMAS-lifting.

The results of the study can underlie the development of objective markers for predicting the effectiveness of surgical interventions performed to correct involuntary changes in the skin system.

## 5 Conclusion

Based on the data presented, the following conclusions can be drawn.

With age, the FRP imbalance grows, which is reflected in the reliable regression of the ICb on the background of the ICs and MDA indicators progression, which correlates with skin microcirculation disorders, manifested by the increasing energy deficit and the severity of involutional skin changes. This justifies the advisability of using conditioning by energy-correcting means (such as cytoflavin, meldonium, etc.). Preoperative pharmacological conditioning allows to reduce the number of early postlifting complications associated with tissue trophism, especially during surface lifting in a quarter.

Based on the study of postoperative catamnesis, self-assessment data and laboratory-instrumental methods of skin system examination in people of different ages, it was revealed that when using SMAS-lifting with a three-level endoscopic-assisted lifting of the lower part of the face, the lowest incidence of complications and the best 5-year effectiveness were established.

## Declarations

### Author contribution statement

Natalia E. Manturova: Performed the experiments; Analyzed and interpreted the data.

Victor A. Stupin: Conceived and designed the experiments; Performed the experiments; Wrote the paper.

Galina O. Smirnova: Performed the experiments.

Ekaterina V. Silina: Analyzed and interpreted the data; Wrote the paper.

### Funding statement

This research did not receive any specific grant from funding agencies in the public, commercial, or not-for-profit sectors.

### Competing interest statement

The authors declare no conflict of interest.

### Additional information

No additional information is available for this paper.

## References

[1] Franceschi C., Garagnani P., Morsiani C., Conte M., Santoro A., Grignolio A., Monti D., Capri M., Salvioli S. (2018). The continuum of aging and age-related diseases: common mechanisms but different rates. Front. Med..

[2] Song S., Johnson F.B. (2018 Apr 9). Epigenetic mechanisms impacting aging: a focus on histone levels and telomeres. Genes.

[3] van de Ven R.A.H., Santos D., Haigis M.C. (2017). Mitochondrial sirtuins and molecular mechanisms of aging. Trends Mol. Med..

[4] Xu X., Wang B., Ren C., Hu J., Greenberg D.A., Chen T., Xie L., Jin K. (2017). Recent progress in vascular aging: mechanisms and its role in age-related diseases. Aging Dis..

[5] Burke K.E. (2017). Mechanisms of aging and development – a new understanding of environmental damage to the skin and prevention with topical antioxidants. Mech. Ageing Dev..

[6] Gorkun A.A., Kozhina K.V., Zurina I.M., Kosheleva N.V., Saburina I.N. (2016). Pathophysiological and molecular mechanisms of extracellular matrix protein resorption during skin aging, and the ways to their restoration. Patol. Fiziol. Eksp. Ter..

[7] Velarde M.C., Demaria M. (2016). Targeting senescent cells: possible implications for delaying skin aging: a mini-review. Gerontology.

[8] Silina E.V., Manturova N.E., Mamedov E.V., Smirnova G.O. (2012). The clinical and laboratory evaluation of the facial skin state after surgical correction. Khirurgiia.

[9] Hassouneh B., Brenner M.J. (2015 Aug). Systematic review and meta-analysis in facial plastic surgery. Fac. Plast. Surg. Clin. N. Am..

[10] Kobus K., Kobus-Zaleśna K. (2018 Jan 16). Remarks on perfection in plastic surgery of the face. Biomed. Res. Int..

[11] Shay P., Taub P.J., Silver L. (2016 Jul–Aug). Improved techniques and future advances in plastic surgery in global health. Ann. Glob. Health.

[12] Boahene K., Brissett A.E., Jones L.R. (2018 May). Facial plastic surgery controversies: keloids. Fac. Plast. Surg. Clin. N. Am..

[13] Lin D.J., Wong T.T., Ciavarra G.A., Kazam J.K. (2017 Nov-Dec). Adventures and misadventures in plastic surgery and soft-tissue implants. Radiographics.

[14] Kozina L.S., Borzova I.V., Arutiunov V.A., Ryzhak G.A. (2012). The role of oxidative stress in skin aging. Adv. Gerontol..

[15] Manturova N.E., Silina E.V., Stupin V.A., Smirnova G.O., Bolevich S.B. (2012). Free radical processes in the pathogenesis of involutional skin changes. Ter. Arkh..

[16] Lephart E.D. (2016). Skin aging and oxidative stress: Equol's anti-aging effects via biochemical and molecular mechanisms. Ageing Res. Rev..

[17] Wölfle U., Seelinger G., Bauer G., Meinke M.C., Lademann J., Schempp C.M. (2014). Reactive molecule species and antioxidative mechanisms in normal skin and skin aging. Skin Pharmacol. Physiol..

